# Case Report: Summary of multiple CAR-T expansions in anti-BCMA/GPRC5D bispecific CAR-T cell therapy for multiple myeloma

**DOI:** 10.3389/fimmu.2025.1607778

**Published:** 2025-06-06

**Authors:** Liya Wei, Xingxian Xiao, Xin Jing, Yuwei Zheng, Xiaoyan Sun, Wei Bai, Manjun Li, Min Luo, Yang Xiao

**Affiliations:** ^1^ Department of Hematology, Shenzhen Qianhai Shekou Pilot Free Trade Zone Hospital, Shenzhen, China; ^2^ Department of Hematology, Jiangmen Central Hospital, Jiangmen, China; ^3^ Department of Intensive Care Unit, Shenzhen Qianhai Shekou Pilot Free Trade Zone Hospital, Shenzhen, China; ^4^ Guangzhou Bio-Gene Technology Co., Ltd., Guangzhou, China

**Keywords:** anti-BCMA/GPRC5D bispecific CAR-T cell therapy, multiple myeloma, extramedullary disease, cytokine release syndrome, expansions

## Abstract

Chimeric antigen receptor (CAR) -T cell therapy targeting B-cell maturation antigen (BCMA) has demonstrated significant efficacy and is considered an ideal target for the treatment of relapsed or refractory multiple myeloma (R/R MM). However, due to the unstable or negative expression of BCMA, single-target BCMA CAR-T cell therapy still faces challenges, whereas targeting G protein-coupled receptor C5 family member D (GPRC5D) provides a new therapeutic direction. Clinical studies have shown that CAR-T cell therapy targeting GPRC5D has promising therapeutic potential for R/R MM. Here, this study is a case report on a 61-year-old male R/R MM patient with extramedullary disease (EMD) who participated in a clinical trial of anti-BCMA/GPRC5D bispecific CAR-T cell therapy. Three months after infusion, the patient achieved a very good partial response (VGPR). Although the patient experienced four episodes of CAR-T cell expansion and developed grade 3 cytokine release syndrome (CRS), the symptoms were well controlled, and the treatment demonstrated generally safe. Our report analyzes the reasons for the four CAR-T cell expansions, highlighting the need for close monitoring and laboratory testing during anti-BCMA/GPRC5D bispecific CAR-T cell therapy. Clinical trial registration: This study was registered on ClinicalTrials.gov, number NCT06068400.

## 1 Introduction

Innovative immunotherapies, particularly chimeric antigen receptor T (CAR-T)cell therapy, have shown potential in treating relapsed or refractory multiple myeloma (1). B-cell maturation antigen (BCMA) is a widely and almost exclusively expressed antigen on plasma cells and B cells, making it a promising therapeutic target for multiple myeloma (2). However, myeloma cells with low to negative BCMA expression may evade BCMA-targeted CAR-T cell therapy and lead to relapse (3). G protein-coupled receptor C5 family member D (GPRC5D) is highly expressed on the surface of primary multiple myeloma cells and is independent of BCMA expression (4). Furthermore, GPRC5D is minimally expressed in bone marrow samples of other hematologic malignancies, making it a promising immunotherapeutic target for treating multiple myeloma patients (5). For multiple myeloma patients who are BCMA-negative or have low BCMA expression, as well as those who experience BCMA-negative relapse due to target cell antigen immune escape after BCMA-targeted therapy, targeting GPRC5D offers a new therapeutic direction (6). A previous clinical study validated the safety and efficacy of anti-GPRC5D CAR T cells (7). Dual-target CAR T-cell therapy can increase the targetable antigens on tumor cells, thereby enhancing long-term therapeutic effects and reducing the incidence of antigen-negative escape (8). Early studies also showed that engineering bispecific CAR-T cells is a promising strategy to overcome the limitations of single-target CAR-T cell therapy and enhance CAR-T cell function (9). Furthermore, a phase 1 clinical study in 2024 demonstrated that anti-BCMA/GPRC5D bispecific CAR T-cell therapy is a promising treatment modality for multiple myeloma, suitable for patients with relapsed or refractory multiple myeloma (R/R MM) (10).

The overall efficacy of CAR-T cell therapy in MM is quite promising, but its toxicity varies from individual to individual, ranging from clinical to life-threatening levels (11, 12). Cytokine release syndrome (CRS) is the most common adverse reaction following CAR-T cell infusion. It has been reported that nearly 46% of B-cell acute lymphoblastic leukemia (ALL) patients receiving anti-CD19 CAR-T therapy and 13% of B-cell lymphoma patients experience severe CRS (≥ Grade 3), while 41% of MM patients develop severe CRS (13). Appropriate CRS is believed to help in tumor cell clearance. However, excessive CRS can lead to significant organ damage and often put patients at risk (14). Previous studies showed that CRS is accompanied by a significant elevation of various cytokines, with IL-6, IL-1β, and IFN-γ mediated by monocyte/macrophage signaling playing a crucial role in the development of the cytokine storm (15).

In this study, a patient with R/R MM treated with anti-BCMA/GPRC5D bispecific CAR-T cell therapy is reported. Although four occurrences of CAR-T cell expansion and the development of grade 3 CRS, the symptom was well controlled. We suggest that multiple factors may collectively trigger CAR-T re-expansion, leading to more severe toxic reactions. This study suggests that CAR-T re-expansion requires closer monitoring and effective medical management to mitigate life-threatening severity.

## 2 Case presentation

M1801 is a 61-year-old male who was hospitalized 4 years ago due to iliac bone pain. He has no underlying diseases or a family history of hematological malignancies (**Table 1**). The patient underwent right frontotemporal approach for malignant cranial tumor resection, duraplasty, cranioplasty. He was diagnosed with MM through positron emission tomography/computed tomography (PET/CT) and bone marrow biopsy pathology, which showed multiple bone destructions. He underwent 4 cycles of PAD regimen chemotherapy (bortezomib, doxorubicin, and dexamethasone) followed by autologous stem cell transplantation, achieving a complete response (CR). After 4 cycles of maintenance therapy with the VD regimen chemotherapy (bortezomib and dexamethasone), the patient’s condition relapsed. He underwent four more cycles of PAD regimen chemotherapy but still experienced a relapse. Subsequently, he underwent radiotherapy, but the disease continued to progress. Next, he received daratumumab combined with selinexor and dexamethasone, but the disease progression remained uncontrolled. Large masses appeared in both iliac bones and gradually increased in size. A follow-up PET-CT indicated extramedullary disease (EMD), with sizes measuring 7.5 cm × 4.0 cm on the left side and 7.9 cm × 2.9 cm on the right side. Given the patient’s recurrent disease after multiple lines and various drug treatments, he was enrolled in a clinical study of anti-BCMA/GPRC5D bispecific CAR-T cell therapy (NCT06068400) (**Table 2**).

**Table 1:** General clinical characteristics of the patient.

Patient information	Patient
Age	61
Sex	Male
Diagnosis	MM IgG(I stage,Lambda Light chain)
Autologous stem cell transplantation	Yes
Bulky disease	Left iliac bone,7.5 × 4.0 cm in size, Right iliac bone, 7.9 × 2.9 in size
Time of initial diagnosis	5 years ago

**Table 2:** Prior therapies of the patient.

Treatment regimen	Efficacy
Right frontotemporal approach for malignant cranial tumor resection, duraplasty, cranioplasty	
PAD (Bortezomib, Doxorubicin, and Dexamethasone)	
Autologous stem cell transplantation	CR
Thalidomide	
VD (Bortezomib and Dexamethasone)	Relapse
PAD (Bortezomib, Doxorubicin, and Dexamethasone)	Relapse
Radiotherapy with GTV_R and GTV_L regimens	Relapse
Daratumumab combined with selinexor and dexamethasone	Relapse

**Figure 1:**
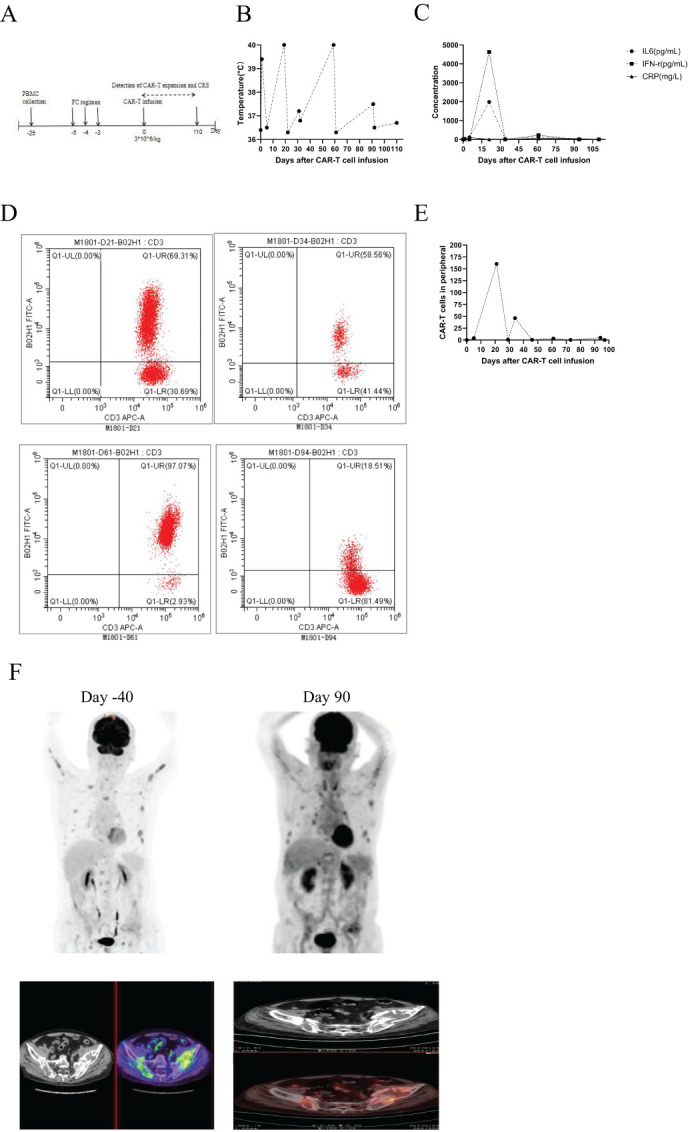
**(A)** Timeline of CAR-T cell therapy, from peripheral blood collection to 3 months post CAR-T cell infusion. **(B)** Changes in body temperature following CAR-T cell infusion. **(C)** Levels of IL-6, IFN-γ, and C-reactive protein in serum at different time points after CAR-T cell infusion. **(D)** Expansion of CAR-T cells in PBMCs at different time points. **(E)** Kinetics of circulating CAR-T cells in M1801, absolute number of CAR-T is shown. **(F)** PET/CT scans revealed a reduction in activity at 3 months post-CAR-T cell therapy, indicating that the patient remains in remission.

M1801 then received preconditioning with the FC regimen chemotherapy (fludarabine 30 mg/m² from day -5 to -3, cyclophosphamide 300 mg/m² from day -5 to -3). The patient received anti-BCMA/GPRC5D bispecific CAR-T cells at a dose of 3 × 10^^^6 cells/kg according to the clinical study protocol (**Figure 1A**). The assessment of CRS follows the ASTCT evaluation criteria (16). The grading and management of CRS follow the recommendations for the assessment and management of CAR-T cell therapy-related toxicities (16, 17). On the first day after CAR-T cell infusion, the patient’s vital signs were stable, but he developed high fever with a temperature of 39.4°C (**Figure 1B**; **Table 3**), which was considered grade 1 CRS combined with infection. Symptomatic treatment for fever and preventive anti-infection therapy were provided. On the fourth day after CAR-T cell infusion, the patient’s transaminases were more than three times the normal value. He was given enhanced liver protection treatment, and tocilizumab was administered to antagonize IL-6. By the fifth day post-infusion, the patient’s temperature returned to normal, and liver function normalized by the seventh day. CAR-T cells begin to expand for the first time. On the 19th day post-infusion, the patient experienced recurrent high fever with a maximum temperature of 40°C, along with chills and rigors, which was assessed as grade 2 CRS combined with infection. Meropenem was administered for infection treatment, along with tocilizumab to antagonize IL-6. On the 21st day post-infusion, the patient developed lactic acidosis, which was assessed grade 3 CRS combined with Hemophagocytic Lymphohistiocytosis (HLH), and dexamethasone was used to block CRS. Meropenem combined with vancomycin was given for infection treatment, and methylprednisolone was administered along with tocilizumab and etoposide to suppress the response. The patient was transferred to the Intensive Care Unit (ICU) for further treatment, where he developed multiple organ failure and severe bone marrow suppression. Treatments included the removal of inflammatory mediators and the correction of acid-base and electrolyte balance, as well as anti-infection and supportive symptomatic treatments. Continuous renal replacement therapy (CRRT) was performed to timely remove inflammatory and acidic metabolic products (18). On the 31st day post-infusion, the second CAR-T expansion occurred, peaking on the 34th day. Methylprednisolone combined with intravenous immunoglobulin was administered to block CAR-T expansion. On the 43rd day post-infusion, the patient was transferred out of the ICU. However, on the 59th day post-infusion, the patient again developed high fever, with a maximum temperature reaching 40°C, and experienced the third CAR-T expansion, peaking on the 61st day. Cyclophosphamide and fludarabine were used to suppress CAR-T expansion. The CAR-T expansion was accompanied by CRS and HLH, and hydrocortisone was used to suppress the inflammatory response. CRRT was performed to remove inflammatory mediators immediately, and the IFN-γ inhibitor imatinib was administered, along with ruxolitinib to block hemophagocytic syndrome. On the 91st day, the patient exhibited low fever with a temperature of 37.5°C, heart failure, and the fourth CAR-T expansion occurred, peaking on the 94th day. Methylprednisolone combined with ruxolitinib was used to alleviate the inflammatory response. The level of CAR-T cells in peripheral blood mononuclear cells (PBMCs) peaked at 69.31% (160.21 cells/uL) on day 21 (**Figures 1D, E**). On day 21 post-CAR-T cell infusion, serum IL-6 peaked at 1981.00 pg/ml, and serum IFN-γ reached a peak of 4626.00 pg/ml (**Figure 1C**). One month after CAR-T cell therapy, flow cytometry showed negative minimal residual disease (MRD) in the bone marrow, negative immunofixation electrophoresis, negative serum M protein, and negative urine M protein. Two months after CAR-T cell therapy (**Table 4**), PET/CT showed reduced activity in the large masses in the hip region. Additionally, pathological examination of the lesions at high metabolism sites under CT guidance showed no activity three months after CAR-T cell therapy (**Figure 1F**). After comprehensive evaluation, the patient achieved very good partial response (VGPR) three months after CAR-T cell infusion. As of 110 days post-infusion, the patient remained in a state of remission.

**Table 3:** Changes in vital signs following CAR-T cell infusion.

Vital signs	D0	D1	D5	D19	D21	D31	D59	D91	D110
Tempemperature (°C)	36.6	39.4	36.5	40	37.8	37.2	40	37.5	36.7
Pulse rate (/min)	96	124	92	132	148	88	80	116	81
Respiratory rate (/min)	20	23	20	25	36	13	22	21	20
Blood pressure (mmHg)	97/68	107/67	104/59	102/70	118/71	129/57	114/69	107/72	126/85

**Table 4:** Treatment efficacy evaluation.

	Before treatment	After treatment
Minimal residual disease	Positive	Negative
Serum immunofixation electrophoresis	IgG(Lambda Light chain)	Negative
Urine immunofixation electrophoresis	IgG(Lambda Light chain)	Negative
Serum M protein	2.88g/L	Negative
Urine M protein	Negative	Negative

**Figure 2:**
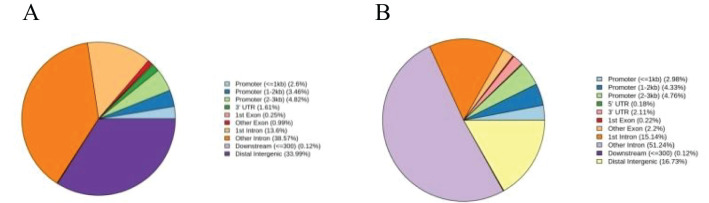
**(A)** Statistical distribution chart of integration sites in gene functional regions in the second expansion of CAR-T cells. **(B)** Statistical distribution chart of integration sites in gene functional regions in the third expansion of CAR-T cells.

Most patients experience only one CAR-T cell expansion, but M1801 experienced four expansions (**Figure 1D**). During the first CAR-T expansion, the patient developed life-threatening CRS that led to multiple organ dysfunction, but he recovered after being promptly transferred to the ICU for rescue. To gain a deeper understanding of the reasons for M1801’s multiple episodes of CRS, we conducted a detailed study of this unique case. The first CRS occurred on day 4 and was quickly alleviated after the administration of tocilizumab. The first CAR-T expansion occurred, liver function impairment (grade 1) was temporary and resolved by day 7. The second CRS appeared on day 18 and was accompanied by severe liver function dysfunction (grade 3). It is considered that cytokines released by CAR-T cells in peripheral blood may cause liver function disturbance. Since CAR-T cells are typically activated by targeted antigens, we speculate that the presence of BCMA/GPRC5D-expressing cells may play a stimulating role. On day 31, the second CAR-T expansion occurred, and high-throughput sequencing of pathogenic microbial nucleic acids in peripheral blood indicated infections with rubella virus and human cytomegalovirus (HCMV). The complete blood count indicates severe marrow suppression, and the lymphocyte subset analysis shows a significantly reduced expression of the NK cell subset. Interestingly, whole-exome sequencing (WES) results of peripheral blood revealed a homozygous mutation in the TET2 gene (**Table 5**). We performed WES again during the third and fourth CAR-T cell expansion, and no differences were observed in the TET2 gene mutation sites. We performed CAR-T cell integration site analysis on peripheral blood during the second and third CAR-T cell expansions (**Figures 2A, B**). The lentivirus-based integration vector showed relatively more integration events in intronic and intergenic regions, with no indication of construct-specific risks.

**Table 5:** Whole-exome sequencing results of the TET2 gene mutation.

Gene _Symbol	HET/HOM	Consequence _type	HGVS.c	Transcript _biotype	HGVS.p
TET2	HOM	Missense _variant	c.5347A>G	Protein _coding	p.Ile1783Val
TET2	HOM	Missense _variant	c.5384A>G	Protein _coding	p.Ile1762Val
TET2	HOM	Missense _variant	c.5384A>G	Protein _coding	p.Ile1762Val

Overall, the efficacy of M1801 CAR-T cell therapy achieved VGPR. Although M1801 underwent four episodes of CAR-T cell expansion, the treatment exhibited generally safe. However, the patient experienced severe CRS with a strong systemic response induced by CRS.

## 3 Methods

### 3.1 Flow cytometry

To detect the expansion of CAR-T cells in peripheral blood, the following antibodies were used: CD45-Alexa Fluor 488, CD3-PerCP5.5, and tEGFR-PE (BD Biosciences, San Diego, USA). CD45/SSC gating strategy was applied to identify PBMCs and further examine anti-BCMA cells. Since these anti-BCMA CAR-T cells were engineered with a CAR gene carrying a truncated human epidermal growth factor receptor (tEGFR), they could be directly detected in this study using CD3-PerCP5.5 and tEGFR-PE antibodies. To detect multiple myeloma cells in the bone marrow, the following antibodies were utilized: CD45-V500, CD19-PE-Cy7, CD20-PerCP-Cy5.5, CD56-PerCP-Cy5.5, CD38-FITC, and CD138-APC (BD Biosciences, San Diego, USA). Multiple myeloma cells were identified based on CD138 positivity and strong CD38 expression. Additionally, in the above flow cytometry analysis, debris was first excluded based on light scatter properties.

### 3.2 Cytokine measurement

The levels of TNF-α, IFN-γ, IL-2, IL-4, IL-6, IL-10, and CRP in serum were measured using commercial ELISA kits (R&D Systems, Minneapolis, USA) and analyzed according to the manufacturer’s instructions using the corresponding experimental protocols.

### 3.3 Whole exome sequencing

Library preparation was performed using the MGIEasy Universal DNA Library Prep Kit (MGI, China). A certain amount of genomic DNA was fragmented, followed by fragment selection. The reaction system was then prepared and programmed to repair DNA ends and add an A base to the 3’ end. Adapter ligation was performed by preparing and programming the adapter ligation reaction system to connect the adapters to the DNA fragments.

Pre-PCR was conducted by preparing the reaction system and setting the reaction program to amplify the product. A specific amount of PCR product was hybridized using the SureSelectXT Human All Exon V6 Kit (Agilent, USA). The adapter-ligated library was hybridized in solution with a biotin-labeled probe library, allowing the probes to bind to the target DNA fragments based on complementary base pairing. Streptavidin magnetic beads were then mixed with the hybridization mixture to firmly bind the beads to the biotin-labeled target fragments, thereby capturing the exonic regions of the genome. Further washing steps were performed to remove nonspecifically bound DNA, enriching the exon-targeted DNA in the library.

A PCR reaction system was then prepared and programmed to amplify the enriched DNA. The PCR product was denatured into single strands, followed by the preparation and execution of a circularization reaction to generate single-stranded circular DNA. Uncircularized linear DNA molecules were digested, yielding the final library. The final library was amplified using phi29 polymerase to generate DNA nanoballs (DNBs). These DNBs were loaded onto a high-density DNA nanochip and sequenced on a G400 sequencer (BGI-Shenzhen, China) using combinatorial probe-anchor synthesis (cPAS) technology with PE150 sequencing.

### 3.4 Lentiviral vector integration site analysis

Genomic DNA was fragmented and processed using the Vazyme VAHTS^®^ Universal DNA Library Prep Kit (VazymE, China) for end repair, A-tailing, and adapter ligation. Primers were designed based on the vector’s LTR/ITR sequences and asymmetric adapter sequences. Two rounds of PCR were performed to enrich the integration sites and ligate Illumina sequencing elements.

## 4 Discussion

Anti-BCMA CAR-T cell therapy became the first cellular immunotherapy approved by the U.S. Food and Drug Administration (FDA) in 2021 for the treatment of multiple myeloma, achieving unprecedented clinical efficacy (19, 20). However, due to the variable expression of BCMA antigen on myeloma cells, some patients fail to achieve a good response (21, 22). A preclinical study demonstrated that CAR-T cell therapy targeting both BCMA and GPRC5D can reduce relapse caused by BCMA antigen escape and eliminate BCMA-negative multiple myeloma cells in a mouse model (23). A previous clinical study has shown that anti-BCMA/GPRC5D bispecific CAR-T cells exhibit good safety and promising activity in patients with relapsed or refractory multiple myeloma (10). The results showed that there was no significant difference in efficacy between patients with single-positive expression of BCMA or GPRC5D and those with double-positive expression. Comparing the efficacy and safety of anti-BCMA/GPRC5D CAR T-cell therapy with existing treatment modalities, the anti-BCMA/GPRC5D bispecific CAR-T cells demonstrated a better safety profile, showing lower grades of cytokine release syndrome compared to BCMA-targeted therapies, but similar to GPRC5D-targeted CAR-T cells in the same center (24).

M1801 was a patient with MM complicated by EMD and was resistant to bortezomib-based chemotherapy. Due to the high tumor burden, MM patients with EMD is associated with a higher risk and poor prognosis, and many treatment methods, including ASCT, have failed to improve patient outcomes in most studies (25). M1801 relapsed after chemotherapy, daratumumab, and autologous hematopoietic stem cell transplantation. Therefore, the patient was enrolled in a clinical trial of anti-BCMA/GPRC5D bispecific CAR-T cell therapy. Although CAR-T cells may undergo re-expansion in certain cases, the reason for CAR-T cell re-expansion has not yet been fully elucidated (26). In this study, we report a rare case in which the patient experienced four consecutive waves of CAR-T cell expansion following infusion.

The first two CAR-T expansions were significant and were accompanied by severe CRS and HLH. To our knowledge, this is not the only documented case to date in which confirmed CAR-T clonal expansion led to severe CRS. We speculate that several factors may have contributable to this unexpected complication. First, EMD is associated with an increased risk of higher-grade CRS (27). Second, the interaction between CAR-T cells and antigen-expressing cells at local lesions may induce severe immune response (28) Third, viral reactivation might have occurred in the setting of immune dysfunction following CAR-T therapy (29). Fourth, a somatic TET2 mutation was likely a potential intrinsic factor in the clonal expansion of CAR-T cells and the subsequent fatal inflammatory storm in this patient, independent of the manufacturing process (30). The absolute numbers of the third and fourth CAR-T expansions were not high, and cytokine levels remained low, likely due to stimulation from extramedullary residual lesions.

The phenomenon observed in M1801 is reminiscent of a reported case of anti-BCMA CAR-T cell therapy (patient RJ-31) (31). RJ-31, a 61-year-old patient, likely had a small T-cell clone carrying a TET2 mutation, which posed a hidden risk before being used for CAR-T manufacturing—leading to the uncontrolled expansion of CAR-T cells derived from autologous lymphocytes upon viral infection. In RJ-31’s case, the genetic defect was considered the primary determinant driving CAR-T clonal proliferation, while viral infection may have acted as a trigger, engaging in antigen stimulation alongside CAR-T cells. Although there are similarities between M1801 and RJ-31 in terms of TET2 mutations and severe toxicity, differences in their subsequent outcomes may be attributed to several factors: First, differences in synergistic effects. While M1801 carried a TET2 mutation, whole-exome sequencing did not indicate significant abnormalities in the TET2 mutation across the four episodes of CRS, and viral infection was also present. In contrast, the TET2 mutation likely played a more critical role in the aggressive expansion of CAR-T cells in RJ-31. However, the other two factors—viral activation and antigen stimulation—should not be overlooked in RJ-31. Second, differences in CRS timing. In M1801, each CRS episode occurred only after the patient had recovered from the previous one, whereas in RJ-31, the next CRS episode occurred consecutively before full recovery from the first inflammatory storm. Third, differences in cytokine profiles. M1801 showed elevations in common inflammatory molecules, while RJ-31 exhibited a markedly expanded and more toxic cytokine signature. However, despite these objective observations, the genetic events that occurred in both M1801 and RJ-31 remain sporadic occurrences.

TET2 mutations are generally considered initiating events for clonal hematopoiesis, and the functional abnormality of TET2 protein can lead to exacerbated immune inflammation, particularly characterized by elevated IL-6 levels (22). The somatic TET2 mutation identified in this study is particularly observed in healthy elderly individuals, where loss-of-function mutations in DNMT3A, TET2, and ASXL1 are among the most commonly detected mutations (32, 33). Before undergoing CAR-T cell therapy, subjects need to undergo WES of peripheral blood. A positive TET2 gene mutation provides important guidance for effectively assessing subsequent treatment risk. We need to further investigate the mechanism by which TET gene mutation triggers CAR-T cell expansion.

Previous study have shown that patients with EMD have higher grades of CRS compared to those without EMD. Additionally, serum IL-6 levels are significantly elevated in patients with EMD. These results may be related to the tumor burden in patients with EMD (34). Therefore, tumor burden should be minimized as much as possible to reduce excessive stimulation of CAR-T cells by target antigen-expressing cells before CAR-T cell therapy.

Before CAR-T cell manufacturing, certain conditions—such as viral infections—may act as antigenic stimuli, leading to ectopic proliferation of CAR-T cells derived from autologous lymphocytes (35). Viral infections can trigger CAR-T cells as part of the adaptive immune response (36). Our study suggests that early administration of antiviral drugs should be considered when using dual-target CAR-T therapy to reduce the risk of severe viral infections (37). The life-threatening CRS observed in this patient highlights the importance of appropriate and timely administration of immunoglobulins or specific antimicrobial agents to minimize the risk of pathogen infection, especially during periods of immunoglobulin decline when circulating CAR-T cells remain in an effector state (38).

In recently reported clinical studies of dual-target CAR-T cells in RR/MM patients, the therapy has shown good clinical activity and safety. We speculate that multiple CRS are not significantly related to dual-target CAR-T cell therapy (24, 39). Additionally, previous studies have shown that among patients receiving CAR-T cell therapy, those who developed CRS had similar clinical outcomes compared to those who did not. There was no significant difference in the complete remission rate or overall response rate between patients who developed CRS and those who did not (40, 41).

During the first CAR-T cell expansion, the patient’s inflammatory cytokines peaked, CRRT was used to remove inflammatory mediators, effectively suppressing the systemic inflammatory response and thereby reducing the resulting organ dysfunction. During the second CAR-T cell expansion, CRRT was applied at an early stage, which prevented severe multi-organ dysfunction. Therefore, if a significant increase in IL-6 is observed during CAR-T cell expansion, early application of CRRT can help prevent multi-organ dysfunction. Additionally, for elderly patients with weakened immunity, the CAR-T cell infusion dose should be appropriately reduced (42). Furthermore, CAR-T cells equipped with suicide genes are highly sought after to mitigate life-threatening side effects (43).

## 5 Conclusion

In summary, our case report discusses the reasons for the four CAR-T cell expansions and provides a treatment summary, deepening our understanding of the systemic toxicity associated with bispecific anti-BCMA/GPRC5D CAR-T cell therapy. Although this study was conducted in patients with R/R MM undergoing treatment, we believe that the findings are not limited to this specific disease type or engineered product. Instead, these conclusions may also apply to other dual-target CAR-T cell therapy. However, due to the limited number of patients in this study, further clinical data are needed to confirm the safety and efficacy of the bispecific anti-BCMA/GPRC5D CAR-T cell therapy.

## Data Availability

The datasets presented in this study can be found in online repositories. The names of the repository/repositories and accession number(s) can be found in the article/supplementary material.
